# The SEPALLATA-like gene *HrSEP1* in *Hippophae rhamnoides* regulates flower development by interacting with other MADS-box subfamily genes

**DOI:** 10.3389/fpls.2024.1503346

**Published:** 2025-01-29

**Authors:** Di Cong, Xue Zhao, Chang Ni, Mengru Li, Luwen Han, Jianlin Cheng, Hongzhang Liu, Huijing Liu, Dan Yao, Shuying Liu, Guoshuang Chen

**Affiliations:** ^1^ College of Life Sciences, Jilin Agricultural University, Changchun, China; ^2^ Guangyuan Food and Drug Inspection and Testing Centre, Sichuan, China; ^3^ Northeast Institute of Geography and Agroecology, Chinese Academy of Sciences, Beijing, China

**Keywords:** *Hippophae rhamnoides*, MADS-box genes, yeast two-hybrid, bimolecular fluorescence complementation, genetic transformation

## Abstract

MADS-box genes are classified into five categories: ABCDE, including *SEP1*, *SEP2*, *SEP3*, *SEP4*, and other homologous genes, which play important roles in floral organ development. In this study, the cDNA sequence of the *HrSEP1* gene was cloned by RT-PCR and confirmed that this gene belongs to the MADS-box gene family. In addition, subcellular localization experiments showed that the *HrSEP1* protein was localized in the nucleus. We verified the interaction of *HrSEP1* with *HrSOC1*, *HrSVP*, and HrAP1 using yeast two-hybrid and bimolecular fluorescence complementation assays. These genes jointly regulate the growth and development of floral organs. We also found a strong synergy between *HrSEP1* and *AP1* genes in sepals, petals, and stamens by transgenic methods and fluorescence quantitative PCR, suggesting that *HrSEP1* and *AP1* may co-regulate the development of these structures. In conclusion, the expression of *HrSEP1* has a certain effect on the development of floral organs, and these findings lay the foundation for further research on the biological functions of MADS transcription factors in *Hippophae rhamnoides*.

## 1 Introduction

The MADS-box gene is a gene that plays a key role in plant growth and development. The name of the MADS-box gene is an acronym for the initials of important transcription factors in four species. M is derived from the yeast MCM1, which plays a regulatory role in cell metabolism, growth, and type (Passmore et al., 1988). A is AG involved in regulating floral organ formation in *Arabidopsis thaliana* (Yanofsky et al., 1990). D is DEF involved in regulating the formation of floral organs of snapdragon (Sommer et al., 1990). S is a human serum response factor SRF, which not only participates in human serum response but also regulates the transcription of proto-oncogene (Norman et al., 1988). They are diverse in type, number, and function and can be classified into two groups based on phylogenetic differences: type I transcription factors and type II transcription factors (Álvarez-Buylla et al., 2000). Among them, type II MADS-box transcription factors dominate, and they all have four specific structural domains: the MADS (M) domain is a conserved region that regulates DNA binding, the spacer domain (I) is involved in the formation of specific DNA-binding dimers, and the keratin (K) has a coiled-coil structure, which is responsible for participating in protein–protein interactions and is currently the validation of protein–protein interaction main site for verification of protein–protein interactions. The most variable region, the C-terminus (C), primarily acts as a transcriptional activator (De Bodt et al., 2003; Gramzow and Theissen, 2010). Based on their structural characteristics, type II genes can be further divided into two subfamilies: MIKCc type and MIKC* type (Kaufmann et al., 2005).

Initially, studies on the function of MADS-box transcription factors in plants focused on the development of floral organs. With the progress of science and research, a large number of MADS-box transcription factors have been found to regulate fruit ripening, softening, and nutrient metabolism in plants as well. In addition, they are involved in the regulation of microbe-induced signal transduction and hormone transport processes (Goto and Meyerowitz, 1994; Mandel and Yanofsky, 1995; Liljegren et al., 2000). By studying the floral organs of plants such as *A. thaliana*, researchers initially proposed the ABC model of the MADS-box gene, which plays a dominant role in the development of plant floral organs (Coen and Meyerowitz, 1991). Over time, more and more MADS-box genes were discovered and cloned in different species, leading to the proposal of the tetramer model and the establishment of the ABCDE model (Saedler et al., 2001; Rijpkema et al., 2010). The calyx is regulated by class A and E genes; petals are controlled by class A, B, and E genes; stamens are governed by class B, C, and E genes; carpels are influenced by class C and E genes; and ovules are modulated by class C, D, and E genes. In the *A. thaliana* genome, four *SEP* (*SEPALATA*) genes have been characterized as *SEP1*, *SEP2*, *SEP3*, and *SEP4*. They exhibit functional redundancy in determining sepals, petals, stamens, and carpels (Ditta et al., 2004). The function of SEP-like genes involves forming complex polymers through protein–protein interactions with class A, B, C, and D genes, acting as “adhesives” that play a crucial role in plant growth processes (Wang et al., 2020).

The class E gene PTM3/4/6 was expressed at all growth stages of male and female flowers in the woody plant *Populus tremuloides* (Pelaz et al., 2000). *In situ*, RNA localization definitively showed that the transcripts of PTM3/4 and 6 were mainly located in the internal sex wheel in the ovule of female flower development and the anther primordium of the male flower. Class E genes are crucial for reproductive growth in woody plants, particularly during flower development. *PmSEP1* and *PmSEP4* are the key genes controlling the development of the plum blossom meristem and sepal. *PmSEP2* and *PmSEP3* play an essential role in the morphogenesis of petals, stamens, and pistils. SEP-like genes regulate plant growth and development together with other MADS-box genes (Zhou et al., 2017). The expression pattern of *PruneSEP1* during the storage of mature soft peaches was similar to that of genes related to ethylene biosynthesis and ethylene signal transduction (*EIN2* and *ETR2*). Furthermore, the expression level of *PruneSEP1* was positively correlated with the expression levels of *EIN2* and *ETR2*. However, this phenomenon did not occur in hard peaches, which proves that *PruneSEP1* regulates the ripening and softening of peaches (Li et al., 2017). Studies have proven that the *SEP* homologous gene *OsMADS34* in rice combines with another gene, *OsMADS1*, which is similar to *SEP* in rice. This specifies the identity of flower organs, including the epidermis/palea, scales, stamens, and carpels (Gao et al., 2010). Patrice Morel et al. definitively showed that the *SEP1*/*SEP2*/*SEP3* homologs of *Petunia*, together with *AGL6*, encode the classic *SEP* floral organ characteristics and flower termination function. The *SEP3* homolog of *Petunia* (*FBP2*) plays a leading role in this process (Morel et al., 2019). The *SOC1* gene is a key regulator of the transition from vegetative to reproductive development in *A. thaliana*. In *Dendrobium nobile*, *DOSOC1* is highly expressed in the inflorescence apex, flower stem, flower bud, and other parts. During the transition period of flowering, *DOSOC1* expression is unequivocally upregulated throughout the entire seedling (Ding et al., 2013). The *SVP* subfamily genes are indisputably linked to the flowering of 1-year species and the dormancy of buds in multi-year species. During the nutritional period, the flower suppressor complex, composed of *SVP* and FLOWERING LOCUS C (*FLC*), definitively downregulates the key flowering gene *SOC1* and flowering site T (FT), thereby inhibiting flowering (Li et al., 2008). The *SEP4* gene plays a role in inflorescence branching in *Arabidopsis* and rice. It can redundantly inhibit *TFL1* with *SOC1* and *SVP* genes. This shows that the *SEP4* gene is involved in regulating inflorescence branching (Liu et al., 2013).

*Hippophae rhamnoides* is a highly beneficial perennial woody plant with numerous advantages for environmental management and human health. The study of anti-pseudorabies virus activity *in vitro* revealed that *H. rhamnoides* polysaccharide can reduce the malondialdehyde content and reactive oxygen species level in cells and increase superoxide dismutase activity. These results prove that *H. rhamnoides* polysaccharide can reduce the oxidative stress caused by pseudorabies virus infection (Huan et al., 2022). Phenylpropanoids extracted from *H. rhamnoides* are an effective treatment for myocardial injury induced by doxorubicin in zebrafish, and they inhibit myocardial apoptosis (Li et al., 2022). *H. rhamnoides* pulp and *H. rhamnoides* oil are extremely rich in bioactive substances and are relatively friendly to the human body. They play a crucial role in the application of traditional Chinese and Tibetan medicine. The inhalation of SO_2_ can change the ratio of liver, lung, kidney, spleen, and other organs in mice’s body. A study proved that rhamnosus seed oil has a protective effect on the damage caused by inhalation of sulfur dioxide in mice (Ruan et al., 2003). *H. rhamnoides* oil is a vital component of cancer treatment during chemotherapy and radiation therapy. It can also help counteract many side effects and assist in treatment, restoring kidney and liver function (Huang et al., 2020). Supplementing with *H. rhamnoides* may affect lipid metabolism in human circulation, but it will not affect blood glucose index and blood pressure value (Geng et al., 2022)^]^.

The MADS-box gene is a crucial pathway for understanding and controlling the developmental mechanisms of *H. rhamnoides*. It can also significantly accelerate progress in plant breeding and crop improvement in biotechnology. Regrettably, there are still only a few reports on the study of MADS-box transcription factors in *H. rhamnoides*. It is, therefore, crucial to improve the regulatory network of MADS-box genes in sea buckthorn and explore the site of action, regulatory mechanism, and interaction mode of MADS-box genes to study the development of *H. rhamnoides* flowers in greater depth. This article establishes the foundation for studying the expression patterns of MADS-box transcription factors in *H. rhamnoides* and the mechanisms of interaction between transcription factors.

## 2 Materials and methods

### 2.1 Plant materials

The Fuxin No. 2 *H. rhamnoides* fruit was taken from the *H. rhamnoides* planting base of Jilin Agricultural University in China. The *H. rhamnoides* fruit was temporarily frozen in a laboratory −80°C ultra-low temperature refrigerator. NC89 wild-type tobacco and 2-week-old *Nicotiana benthamiana* seedlings were used, both of which are stored in laboratory seed management libraries.

### 2.2 RNA extraction and *HrSEP1* gene cloning

Extract RNA using the RNA Pure Plant Kit (CoWin Biosciences, Cambridge, MA, USA; CW0559). We used agarose gel electrophoresis and nanometer titration electrophoresis to detect the quality of the RNA. We extracted the RNA using the RNA Pure Plant Kit (CoWin Biosciences, CW0559). We then detected the quality of the RNA using agarose gel electrophoresis and a NanoDrop ONE (Thermo Scientific, Shanghai, China). We designed gene-specific primers using Primer 7.0 (**Supplementary Table S1**). The *SEP1* gene sequence of *H. rhamnoides* is sourced from the *H. rhamnoides* transcriptome database, which is preserved by the research team (login number: PRJNA612989). We amplified the target gene fragment using PCR technology (Takara, Tokyo, Japan) and named it *HrSEP1*. We used the DNA recovery kit (TIANGEN, Beijing, China; DP209) to recover and purify the target fragment, which was then cloned onto the pMD-18T vector (Takara, 6011).

### 2.3 Bioinformatics analysis of *HrSEP1* gene

The MEGA 10.0 software was used to construct a MADS-box gene phylogenetic tree. The prediction results of the tertiary structure of the *HrSEP1* gene can be obtained through https://swissmodel.expasy.org/interactive. The information related to the *HrSEP1* gene signal peptide can be obtained from http://www.cbs.dtu.dk/services/SignalP/Obtain.

### 2.4 Construction of subcellular localization vectors and infection of *N. benthamiana*

The subcellular localization vector pCAMBIA1300-GFP was cleaved into linear fragments using restriction endonucleases *Bam*HI and *Sal*I. Specific primers with corresponding cleavage sites were designed (**Supplementary Table S1**) to construct the recombinant plasmid pCAMBIA1300-*HrSEP1*. The recombinant plasmid was transformed into *Agrobacterium tumefaciens* competent cell EHA105 and injected into tobacco leaves from the back wound to infect the plant. We observed fluorescence with green fluorescence protein (GFP) as the nuclear marker (Liu et al., 2019).

### 2.5 Construction of yeast two-hybrid vector and yeast cell transformation

Restriction endonucleases *Eco*RI and *Bam*HI were used to cleave the bait protein vector pGBKT7 linearly and design specific primers with corresponding cleavage sites (see **Supplementary Table S1**). The double enzyme digestion experiment was followed by the use of T4 ligase to connect the enzyme digestion product with *HrSEP1*, thus constructing the recombinant plasmid pGBKT7-*HrSEP1*. The plasmid was then transferred into yeast-receptive cell AH109 to confirm that *HrSEP1* has self-exciting activity in yeast cells by observing the color of the strain.

The *H. rhamnoides* MADS-box genes *HrAP1*, *HrSOC1*, and *HrSVP* were screened out from the transcriptome database and combined with the prey protein vector empty pGADT7 to form recombinant plasmids pGADT7-HrAP1, pGADT7-*HrSOC1*, and pGADT7-*HrSVP*. Once the prey protein vector was successfully constructed, the combination of pGADT7-HrAP1+pGBKT7-*HrSEP1*, pGADT7-*HrSOC1*+pGBKT7-*HrSEP1*, and pGADT7-*HrSVP*+pGBKT7-*HrSEP1* were used to cotransfect AH109 yeast receptive cells. The positive control pGADT7-T+pGBKT7-53 and negative control pGADT7-T+pGBKT7-lam were also transformed. Each group of the bacterial solution was applied to solid culture media of SD/-Leu/-Trp/-His/-Ade. The plates were sealed and incubated upside down at 29°C–30°C for 3–5 days.

### 2.6 Construction of bimolecular fluorescence complementary vector and infection of *N. benthamiana*

We used restriction endonucleases *Sma*I and *Xba*I to cleave the BiFc vector pXY104 linearly. Based on the two restriction sites, we designed homologous arm primers to connect the vector pXY104 and *HrSEP1*, forming the recombinant plasmid pXY104-*HrSEP1*. Similarly, we cleaved the BiFc complementary vector pXY106 into linear fragments using restriction endonucleases *Sma*I and *Bam*HI. We designed homologous arm primers to connect the vector pXY106 and the prey gene, combining the prey protein gene sequence and pXY106 sequence, to construct recombinant plasmids pXY106-HrAP1, pXY106-*HrSOC1*, and pXY106-*HrSVP*.

The bacterial solution containing pXY104-*HrSEP1* was mixed with the bacterial solution containing pXY106-HrAP1, pXY106-*HrSOC1*, and pXY106-*HrSVP* recombinant complementary vectors. Then, using a sterile syringe, the mixed bacterial solution was extracted and injected into the wound on the back of the tobacco leaf. The tobacco was left in a dark and ventilated location for 40–70 hours. After cutting the leaves, yellow fluorescent protein (YFP) was used as the nuclear marker for fluorescence detection (Kerppola, 2008).

### 2.7 Construction of plant expression vectors and NC89 tobacco transformation

The plant expression vector pCAMBIA3301 was cleaved into a linear plasmid with a sticky end using restriction endonucleases *Nco*I and *Bgl*II. *HrSEP1* was linked to the linear plasmid pCAMBIA3301 using T4 ligase to form a recombinant plasmid, pCAMBIA3301-*HrSEP1*. The recombinant plasmid was transformed into *A. tumefaciens* competent cell EHA105 and then infected with NC89 tobacco leaves using the leaf disc transformation method (An et al., 2021).

### 2.8 Real-time fluorescence quantitative analysis

The melting curve was definitively established to confirm the specificity of the amplified fragment. The expression data were normalized using actin genes as reference genes. The relative expression of genes was calculated using the 2^−ΔΔCT^ method. All experiments were repeated three times independently.

### 2.9 Statistical analysis

All samples in the experiment were assessed in at least three independent biological replicates, and all data are expressed as the mean ± standard deviation (SD). The t-test or one-way ANOVA with a 5% significance level was used to determine whether there were statistically significant differences between the means. The graphs were constructed using Origin 9.5.1 (Microcal Software Inc., Northampton, MA, USA).

## 3 Results

### 3.1 Analysis of gene sequences

There are 49 MADS-box genes annotated by the Kyoto Encyclopedia of Genes and Genomes (KEGG) pathway in the transcriptome database differential genes, protein analysis of these MADS-box genes found that only 27 of them have relatively complete open reading frames, four of them do not have k-box structural domains specific to MADS-box genes, and analysis of the remaining 23 genes revealed that the proportion of class E genes, SEP, was the largest. After analyzing the remaining 23 genes, it was found that the proportion of SEP in the E-class genes was the largest, and one of them has the most significant difference in expression among all the MADS-box genes. Therefore, we chose this gene as the target gene for the subsequent experiments and named it *HrSEP1*.

We extracted total RNA from rhamnosus fruits and successfully obtained the cDNA sequence of *HrSEP1* by reverse transcription. This allowed us to investigate the relationship between *HrSEP1* and floral organ development. PCR amplified the *HrSEP1* gene sequence. Gene sequencing and DNAMAN V6.0 multiplexing showed that the *HrSEP1* sequence was 735 bp and encoded 244 amino acids.

**Figure 1:**
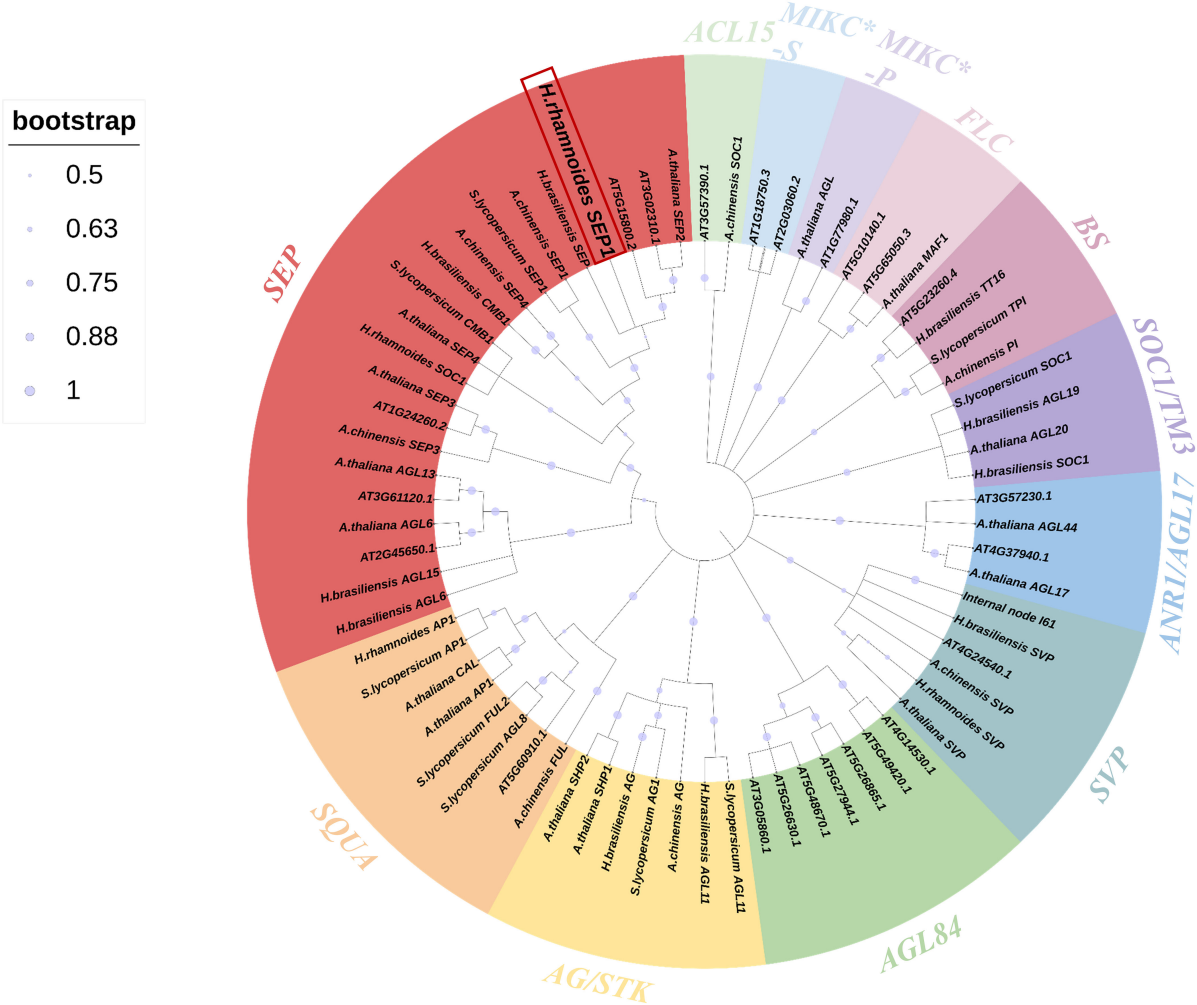
Phylogenetic tree of the MADS-box gene in *Hippophae rhamnoides* L. and the MADS-box genes in *Arabidopsis thaliana*, *Actinidia chinensis Planch*, *Hevea brasiliensis*, and *Solanum lycopersicum L. Arabidopsis* gene sequences from The Arabidopsis Information Resource (TAIR) database; other gene sequences were obtained from the National Center for Biotechnology Information (NCBI) database. Different subfamilies were marked with specific colors. Trees were constructed using the neighbor-joining method, and 1,000 bootstrap replications were performed using MEGA 7.0 International software.

Next, to analyze the function of *HrSEP1* in the MADS gene family, we downloaded MADS gene protein sequences from the National Center for Biotechnology Information (NCBI) database for 70 species, including *A. thaliana*, actinomycetes, neem, and tomato plants. We constructed a phylogenetic tree for comparative analysis. The data clearly show that the *HrSEP1* gene is highly homologous to the SEP genes in *Hevea brasiliensis* and *A. thaliana* and belongs to class E (**Figure 1**).

### 3.2 *HrSEP1* proteins are localized in the nucleus

**Figure 2:**
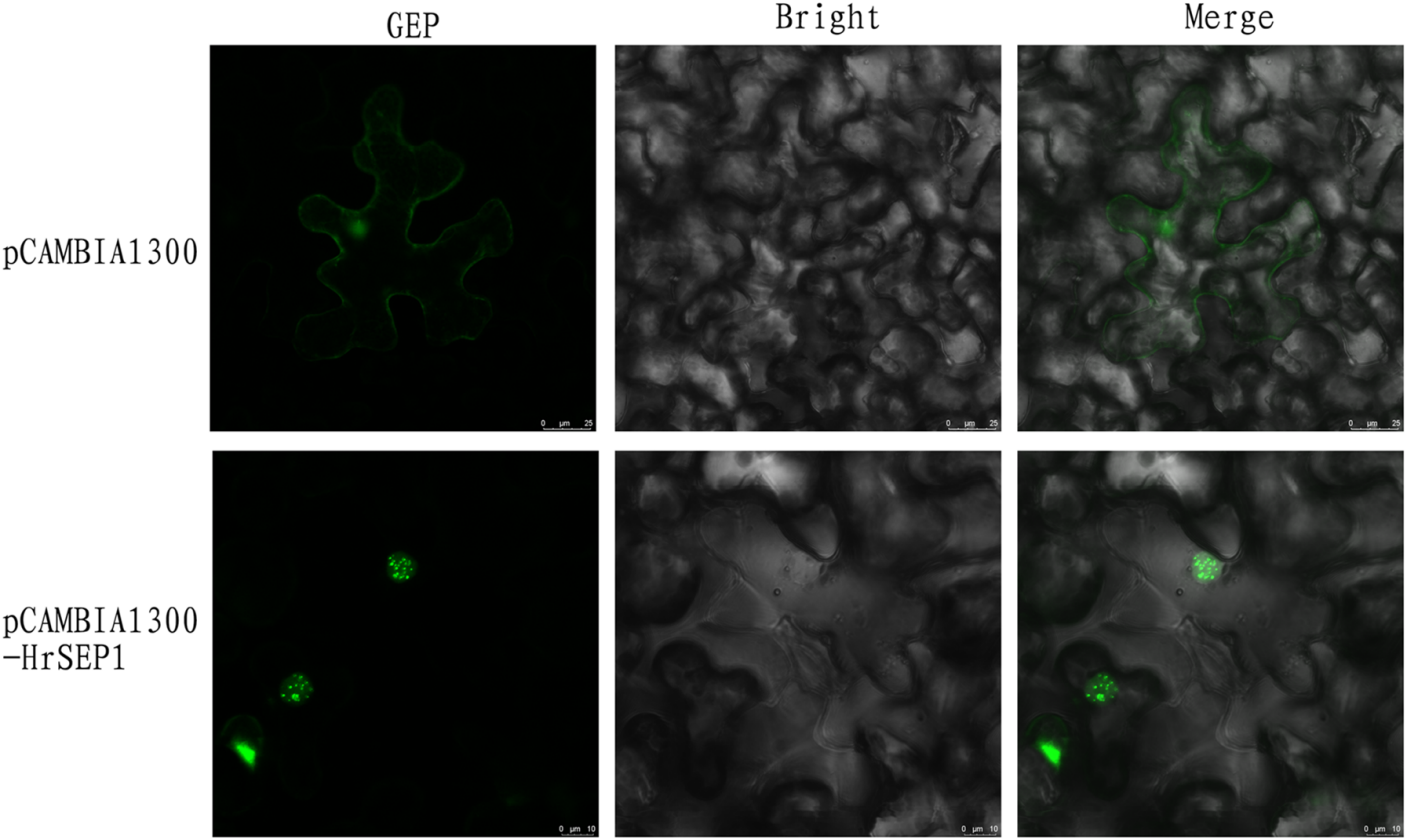
Subcellular localization of *HrSEP1*. Combining *HrSEP1* gene with fluorescent protein vector pCAMBIA1300-GFP can determine the target gene expression location. Green fluorescence protein (GFP) as nuclear marker.

To better understand the function of *HrSEP1*, the *HrSEP1* protein was localized *in vivo*. GFP was fused to the C-terminal of the *HrSEP1* under the control of the CaMV 35S promoter and transiently expressed in *N. benthamiana* leaf epidermal cells, and the results showed that *HrSEP1* is a nuclear-expressed protein (**Figure 2**).

### 3.3 Heterologous expression of *HrSEP1* gene in tobacco

**Figure 3:**
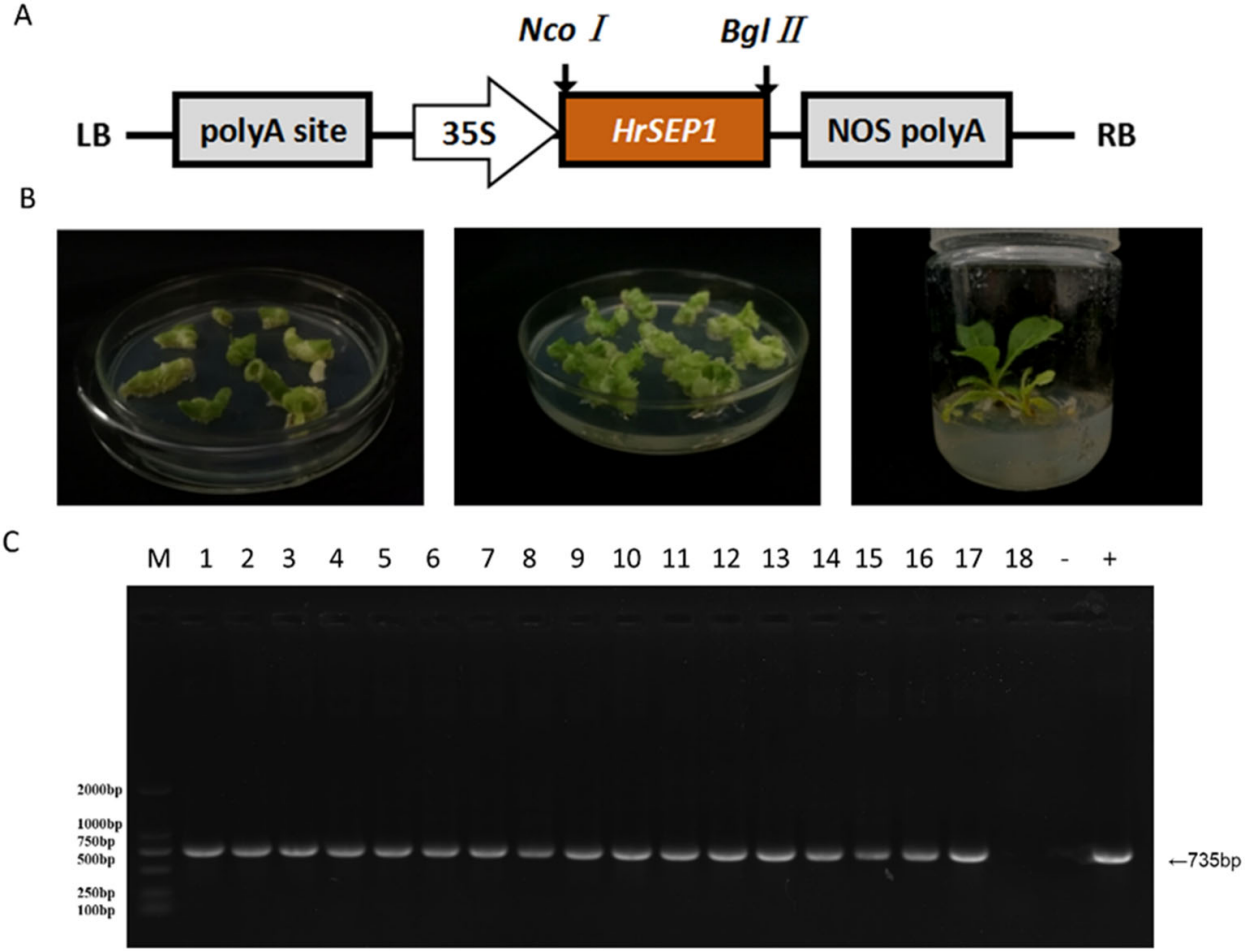
Plant expression vectors were constructed, and T-*HrSEP1* genomic DNA was detected after genetic transformation of tobacco to verify the successful transfer of the *HrSEP1* gene into tobacco. **(A)** Schematic diagram of plant overexpression vectors. **(B)** Transgenic tobacco was obtained using the leaf disc method; from left to right, co-cultures, screening cultures, and differentiation cultures. **(C)** Detection of T-*HrSEP1* genomic DNA in the T-*HrSEP1* genome by PCR: M, DL2000Marker; 1–18, T-*HrSEP1* genomic DNA. *HrSEP1* gene: M, DL2000Marker; 1–18, T-*HrSEP1* genomic DNA; −, negative control; +, positive control.

We constructed a plant overexpression vector, pCAMBIA3301-*HrSEP1* (**Figure 3A**), using pCAMBIA3301 as the vector skeleton, and transformed the recombinant plant expression vector into *A. tumefaciens* competent cell EHA105. We used the leaf disc transformation method to infect NC89 tobacco leaves (**Figure 3B**) using Basta herbicide as a preliminary screen for transgenic plants. We successfully detected the genomic DNA of transgenic plants by PCR and ultimately obtained 17 T2-generation overexpression-positive tobacco plants (**Figure 3C**), which we have named T-*HrSEP1*.

### 3.4 Expression patterns of *HrSEP1* gene regulating flower development-related gene

To analyze the potential function of the *HrSEP1* gene, we verified the expression of known key genes of plant flower development in four organs of the transgenic plant T-*HrSEP1* and the wild-type (WT) plant by qRT-PCR. These key genes are *AP1* (APETALA 1), which is related to floral organ development, *SOC1*, and *SVP*, which are involved in the regulation of flowering time.

**Figure 4:**
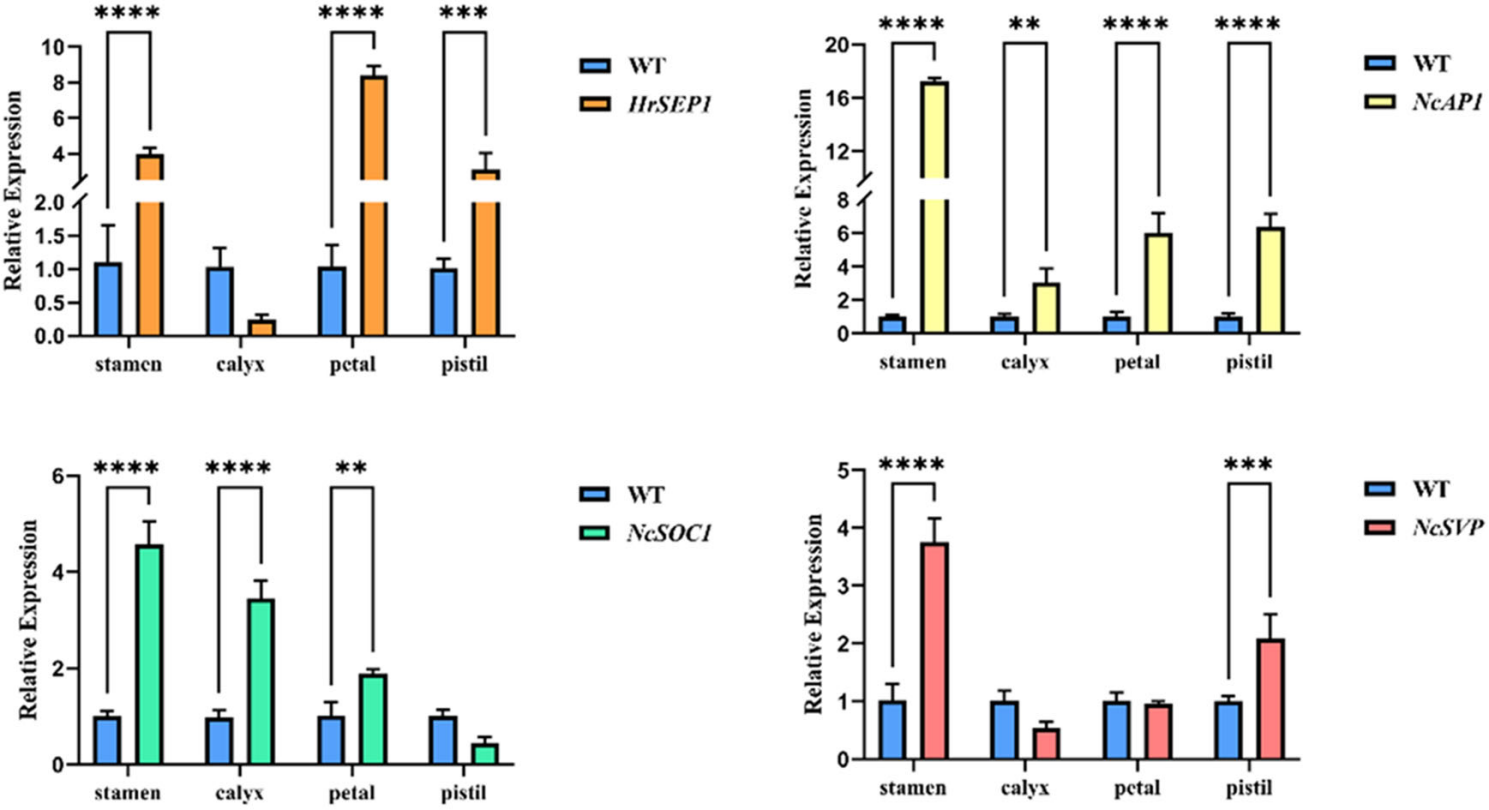
Expression analysis of floral development-related genes *HrSEP1*, *NcSOC1*, *NcSVP*, and *NcAP1* in different parts of floral tissues (including petals, sepals, pistils, and stamens) in transgenic tobacco. Significant differences between each group of data were determined by Student’s t-test (**: 0.001 < p < 0.01; ***: 0.0001 < p < 0.001; ****: 0.00001 < p < 0.0001).

The expression level of *HrSEP1* in the stamens, pistils, and petals of T-*HrSEP1* was significantly higher than that of WT, especially in petals. Conversely, the expression level of *HrSEP1* in the calyx was significantly lower than that of WT (**Figure 4**). The expression of NcAP1 was significantly upregulated in all four floral organs of T-*HrSEP1*, with the highest expression level observed in stamens (**Figure 4**). The expression pattern of *NcSVP* in the calyx of T-*HrSEP1* is contrary to that in other parts, showing a downward trend. However, there is no difference in the expression in petals compared with WT (**Figure 4**). The expression of *NcSOC1* in the pistil of T-*HrSEP1* decreased significantly, while it increased markedly in other parts (**Figure 4**).

These results prove that *HrSEP1* plays a direct or indirect role in the development of tobacco flowers. It does this by regulating the expression of key genes that control flower development.

### 3.5 *HrSEP1* gene is involved in flower development

**Figure 5:**
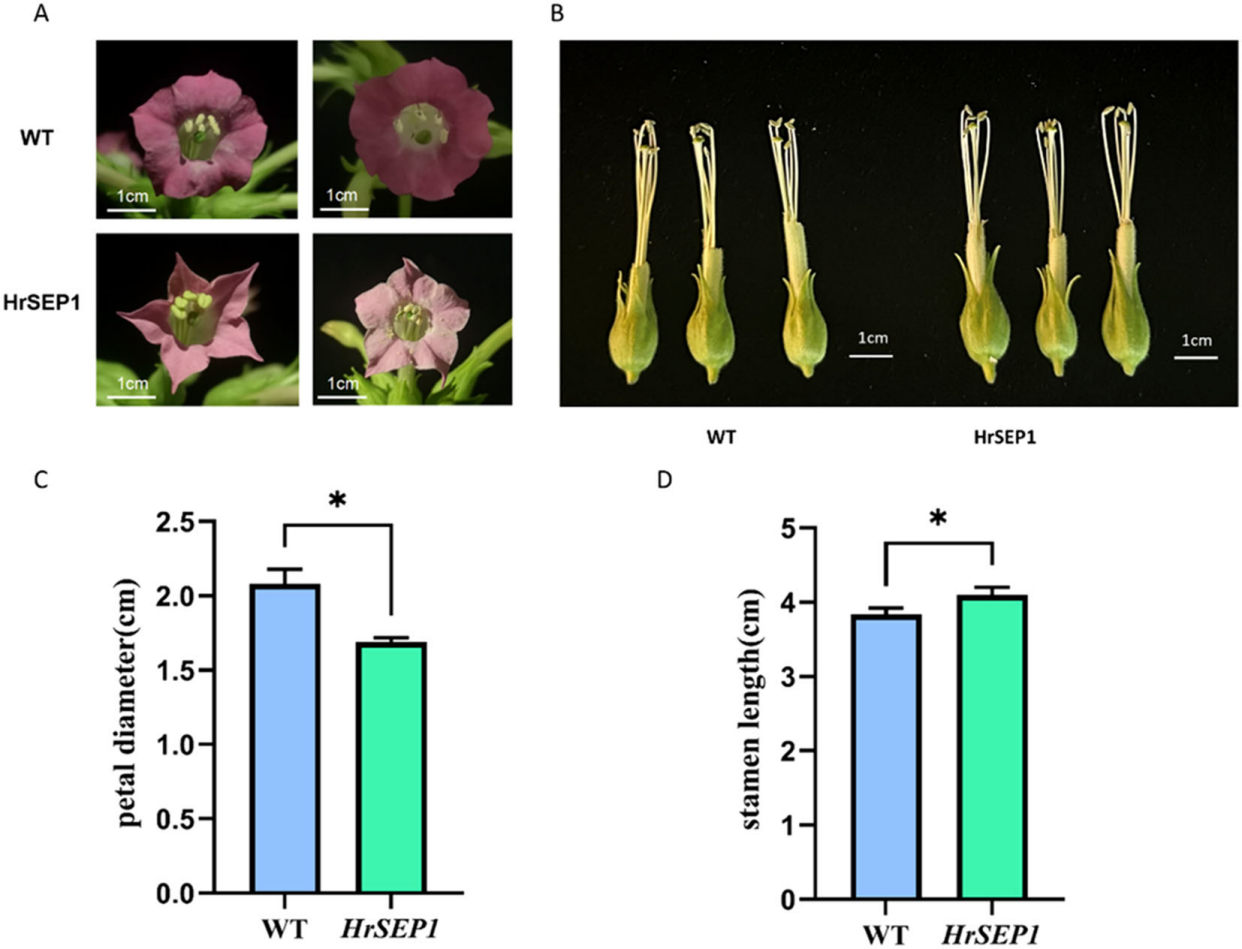
Phenotypic observation of transgenic plants. The scale has a score of 1 cm. **(A)** Comparison of petal shape and color between transgenic tobacco and wild tobacco. **(B)** Comparison of stamen size between transgenic tobacco and wild tobacco. **(C)** Comparison of petal diameters. **(D)** Comparison of stamen lengths. Significant differences between treatment means were determined by Student’s t-test (*, p < 0.05).

Petal development is directly linked to the *SEP* gene. In this study, we randomly selected blooming flowers from three transgenic lines and wild-type plants and observed the color and flower shape of each flower. The flowers of WT were dark purple, while the flowers of T-*HrSEP1* were lighter and pale pink. The petals of WT were rounded, while those of T-*HrSEP1* were sharp and angular. The petal diameter of T-*HrSEP1* was found to be significantly smaller than that of WT (**Figures 5A, C**). qRT-PCR results clearly showed that in plants overexpressing *HrSEP1*, the expression levels of *HrSOC1*, *HrSVP*, and *HrAP1* in the stamen were significantly increased (**Figure 4**). We therefore compared the stamen length of WT and T-*HrSEP1* and found that the stamens of T-*HrSEP1* are slightly longer than those of WT (**Figures 5B, D**). These results demonstrate that *HrSEP1* can affect plant phenotypes by regulating the expression of genes such as *SOC1*, *SVP*, and *AP1*.

### 3.6 *HrSEP1* gene interacts with genes related to flower development

In previous studies, we found significant changes in the expression levels of *AP1*, *SOC1*, and *SVP* genes in tobacco plants overexpressing the *HrSEP1* gene compared with the wild type.

**Figure 6:**
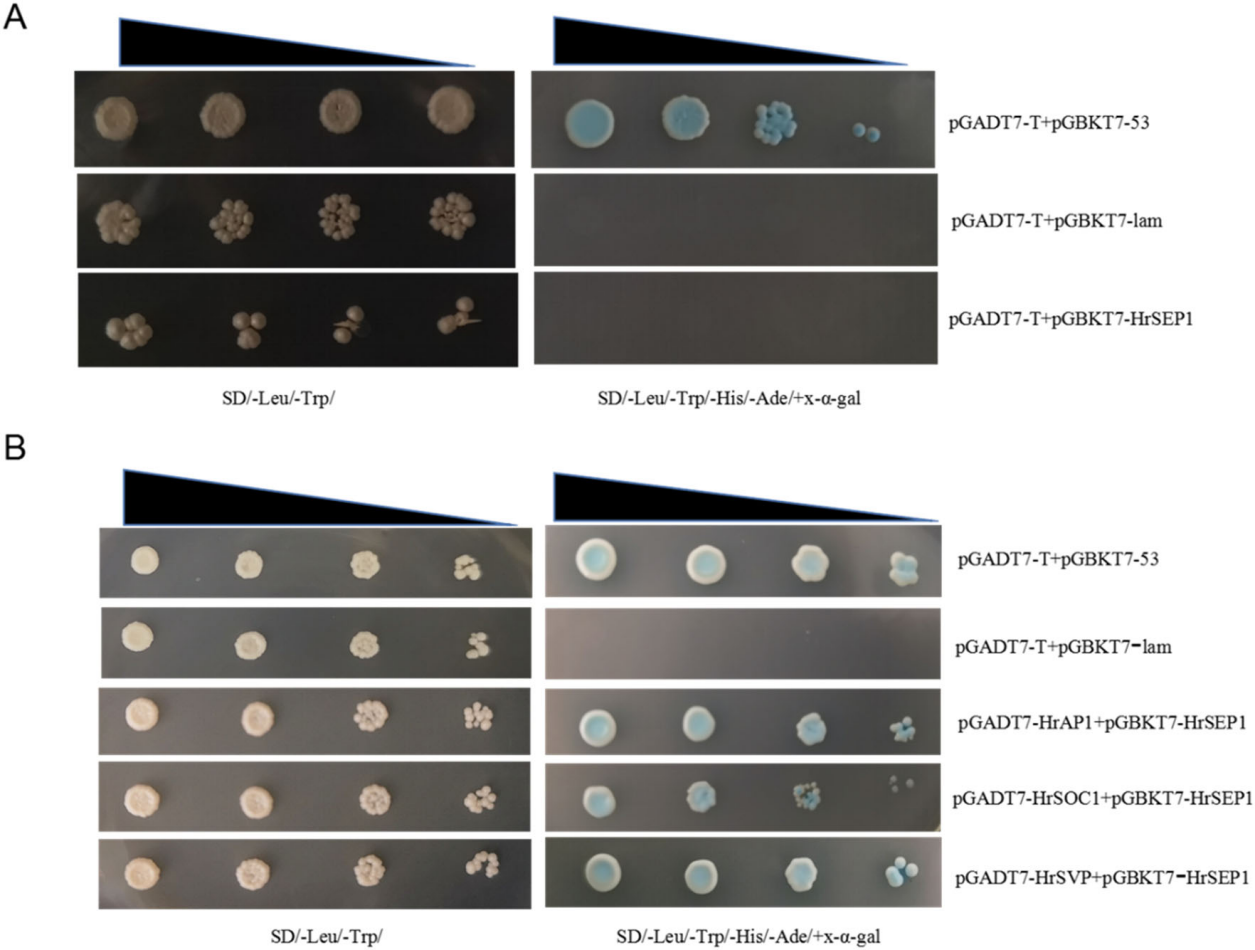
Interaction between *HrSEP1* and *HrAP1*, *HrSOC1*, and *HrSVP* in yeast-competent cells. The interaction is indicated by the growth of blue plaque on SD/Leu/-Trp/-His/-Ade/nutrient-deficient medium. **(A)** Yeast self-activation experiment verifies that *HrSEP1* does not have self-activation activity. **(B)** Yeast two-hybrid experiment to verify the interaction between *HrSEP1* and three other genes.

We successfully constructed prey vectors pGADT7-*HrSOC1*, pGADT7-*HrSVP*, pGADT7-HrAP1, and bait vector pGBKT7-*HrSEP1*, and we verified that the *HrSEP1* gene does not exhibit self-excited activity in yeast receptive cells (**Figure 6A**). This proves that *HrSEP1* does not interact with several other genes. Next, we transformed the yeast-competent cell AH109 with the combination of pGADT7-HrAP1+pGBKT7-*HrSEP1* and spread it on SD/-Leu/-Trp/-ade/auxotrophic medium coated with x-α-gal. We repeated this process with pGADT7-*HrSOC1*+pGBKT7-*HrSEP1* and pGADT7-*HrSVP*+pGBKT7-*HrSEP1*. The results demonstrated that all three combinations could grow on SD/-Leu/-Trp/-His/-Ade/nutrient-deficient medium and produce an obvious blue plaque (**Figure 6B**). This proves that *HrAP1*, *HrSOC1*, and *HrSVP* all interact with *HrSEP1*.

**Figure 7:**
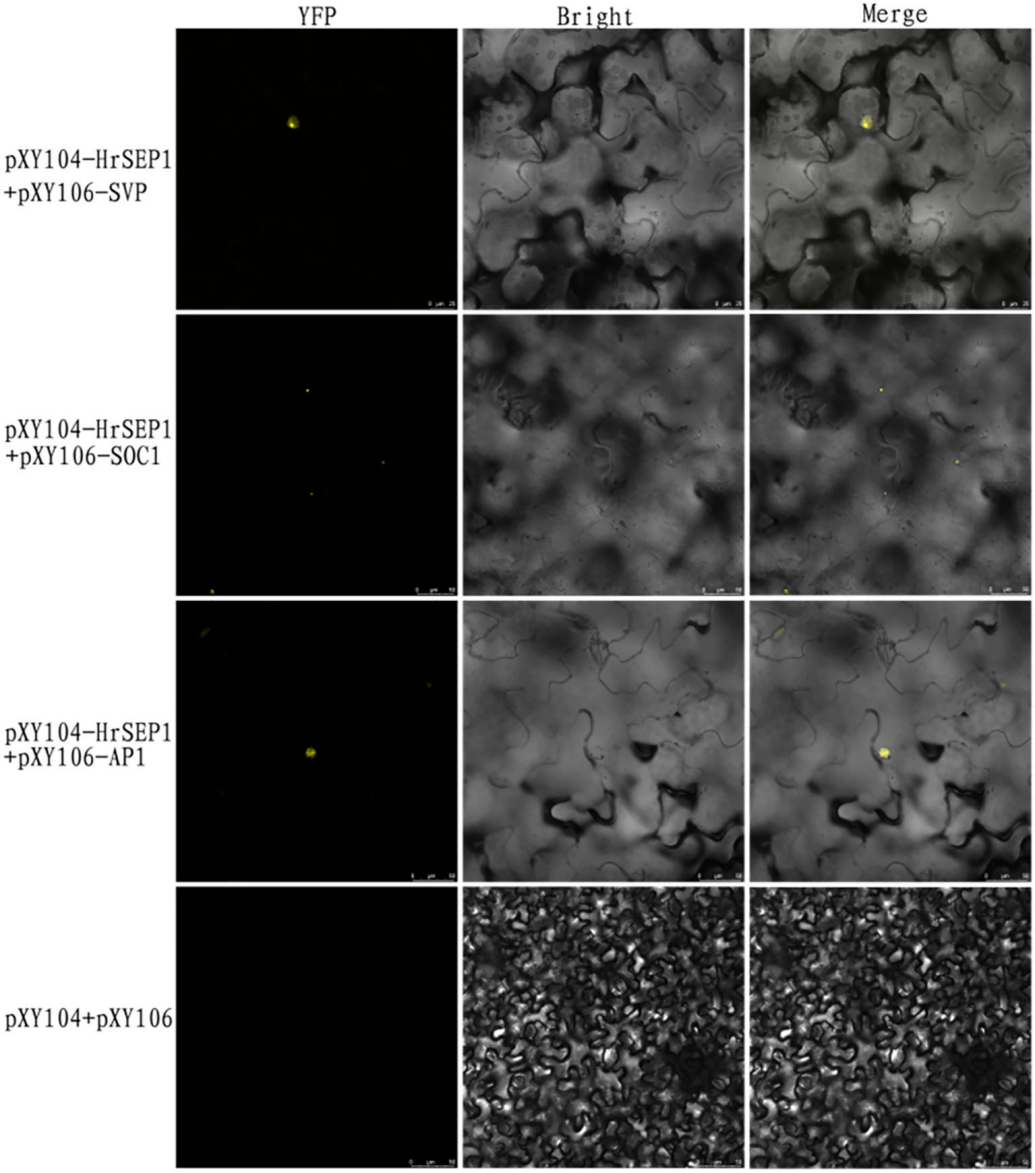
The fluorescence expression of three interaction combinations in *Nicotiana benthamiana* leaves was detected by bimolecular fluorescence complementary experiment to clarify the interaction relationship. Yellow fluorescent protein (YFP) as nuclear marker.

To further verify the interactions between several genes, we constructed bimolecular fluorescent complementary vectors pXY104-*HrSEP1*, pXY106-HrAP1, pXY106-*HrSOC1*, and pXY106-*HrSVP* using pXY104 and pXY106 as vector skeletons. We used the combination of pXY104-*HrSEP1*+pXY106-HrAP1 to co-transform *A. tumefaciens* EHA105 and infect tobacco leaves with the injection method. We performed the same operations on the two combinations of pXY104-*HrSEP1*+pXY106-*HrSOC1* and pXY104-*HrSEP1*+pXY106-*HrSVP*. Observation of YFP fluorescence demonstrated that *HrSEP1* interacts with three genes, *HrSOC1*, *HrSVP*, and HrAP1, in tobacco leaves (**Figure 7**).

## 4 Discussion

The MADS-box gene family is widespread in animals, plants, and fungi, but the number and type of MADS-box genes vary significantly among species. Studies have shown that the number and type of MADS-box genes are more abundant in higher plants, with 108 MADS-box genes in *A. thaliana* and 168 in tobacco (B. et al., 2019; Abdullah-Zawawi et al., 2021). In recent years, MADS genes have also been studied more intensively. A total of 93 PfMADS genes have been identified in the *Perilla* genome. It was further verified that PfMADS47 could effectively mediate the regulation of lipid synthesis by *Chlamydomonas* transformants (Liang et al., 2024). Zhao et al. investigated the MADS-box family in sea buckthorn by conducting a genome-wide survey and expression profiling. They found that 92 MADS-box genes were identified in the *H. rhamnoides* ssp. *sinensis* genome. These genes were distributed on 12 chromosomes and categorized as type I (42) and type II (50). In this study, we identified the MADS-box in sea buckthorn and cloned the *HrSEP1* gene. We analyzed its structural domain, and we constructed a phylogenetic tree. However, the sea buckthorn MADS-box gene family remains to be discovered.

Flower organs are the key to flowering plants. Complex genetic networks control them. The MADS-box gene is the main element in the genetic network that controls the formation of floral organs during plant development. The SEP-like genes of MADS-box genes are crucial for regulating flower transformation and floral organ differentiation (Theissen et al., 2016). The constitutive expression of *IiSEP2* isolated from *Isatis indigotica* in *Arabidopsis* unquestionably leads to changes in inflorescence structure. Overexpression of *IiSEP4* in transgenic *Arabidopsis* restores petal differentiation ability (Ma et al., 2022). In *Habenaria radiata*, the lack of *SEP* function definitively results in a sudden change in the color of orchid flowers to green (Mitoma and Kanno, 2018). The SEPALLATA (SEP) MADS-box gene SlMBP21 negatively regulates the transition from SIM to floral meristem (FM) in the development of tomato sympodial meristem (SIM). Silencing of gene *FaMADS9*, similar to *SEP1/2*, in strawberries definitively inhibits petal development and maturation of the receptacle (Seymour et al., 2010). Overexpression of the marigold gene *TeSEP4* unquestionably leads to a decrease in the number of petals and stamens in transgenic tobacco plants. Conversely, overexpression of *TeSEP1* in tobacco results in longer sepals and simpler inflorescence structures (Zhang et al., 2021). The *SEP3*-like gene *ZaMADS70* interacts with the AP3-like gene *ZaMADS48* in *Zanthoxylum armatum*, promoting petal loss (Tang et al., 2022). These studies prove that MADS-box and *SEP* genes significantly impact plant petals. This study demonstrates that the color and shape of the petals of transgenic tobacco overexpressing the *HrSEP1* gene change, and the stamens also elongate. This result corroborates the findings of the literature review, confirming that *SEP* genes regulate plant petal development. Furthermore, in our study, we found that overexpression of *HrSEP1* significantly increased the expression of the *AP1* gene in tobacco petals, calyx, stamens, and pistils, especially in sepals and stamens, suggesting that *SEP1* and *AP1* may work together in these areas and be involved in their regulation, whereas most studies on the interactions between *SEP1* and *AP1* have focused on the effects on sepals and petals (Yang et al., 2023), with little mention of their synergistic effects in stamens as well. Sepals and petals and their synergistic effect in stamens have rarely been mentioned. In this study, there was a significant increase in *SVP* and *SOC1* gene expression in overexpressing plants, suggesting that they interact. In previous studies, both *SVP* and *SOC1* genes affected flowering time. In *Arabidopsis*, *SVP* is widely expressed during the nutritional development of leaves and stem tips. It works with FLC to negatively regulate SOC1 and FT, inhibiting flowering (Willmann and Poethig, 2011). The overexpression of the *MtSVP* gene isolated from *Medicago truncatula* definitively leads to delayed flowering in *Arabidopsis*. Additionally, flowers exhibit a multitude of abnormal phenotypes, including multi-leaf sepals, changes in the number of floral organs, and longer stems than the wild type. Meanwhile, overexpression of *MtSVP1* unquestionably alters the development of cut-stem alfalfa flowers but does not affect flowering time (Jaudal et al., 2014). Studies have proven that temperature-dependent differential interactions between *SVP* and *FLM* isoforms modulate the temperature-responsive induction of flowering in *Arabidopsis* (Suhyun Jin, 2022). However, this phenomenon was not found in our study, which needs to be further investigated.

E-class genes can form complexes with A-, B-, and C-class genes to jointly command flower organ development. E-class genes can also interact with *SVP* subfamily genes (Tsai and Chen, 2006). Yeast two-hybrid experiments have shown that in tomatoes, *SlMBP21* and *SlMADS1*, genes belonging to the *SEP* branch, can synergistically regulate tomato sepal development by interacting with other regulatory proteins (Zhang et al., 2024). Yeast three-hybrid analysis shows that in the ternary complex, class B proteins can interact with *TeSEP3* by forming a heterodimer *TePI TeAP3-2* (Zhang et al., 2021). Yeast two-hybrid analysis showed that *Platanus acerifolia*’s *PlacSEP* protein can form homologous or heterodimeric dimers with *Platanus APETALA1 (AP1)/FRUITFULL (FUL)*, B-, C-, and D-class MADS-box proteins in different interaction modes and intensities, playing important and different roles in flower initiation and development as well as fruit development (Zhang et al., 2017). Through bimolecular fluorescence complementary (BiFC) analysis, it has been confirmed that the C-class gene *PMADS3* of *Petunia hybrida* plays a dual role in controlling the characteristics of internal organs and the termination of flower meristem tissue. The expressed protein can interact with the E-class proteins *FBP2*, *FBP5*, *FBP9*, and *PMADS12* alone (Li et al., 2015). In this study, we used yeast two-hybrid and bimolecular fluorescence complementation techniques to identify the interaction of the *HrSEP1* gene with *HrAP1*, *HrSOC1*, and *HrSVP*, resulting in changes in plant phenotypes. So far, there has been little research on the role and function of *H. rhamnoides SEP* genes, indicating that there is still considerable research space for the regulatory network mechanism of *H. rhamnoides SEP* genes.

Our study demonstrated that the expression levels of *AP1*, *SOC1*, and *SVP* all exhibited significant changes with overexpression of the *HrSEP1* gene. Through protein interactions, we definitively revealed that the *HrSEP1* gene interacts with the other three MADS genes. These results suggest that *HrSEP1* may have the involvement of *HrAP1*, *HrSOC1*, and *HrSVP* in the regulation of flower development.

## 5 Conclusions

In summary, we obtained an *HrSEP1* overexpression line through a genetic transformation of tobacco. Our qRT-PCR results clearly showed that overexpression of *HrSEP1* had a significant effect on the expression levels of *HrAP1*, *HrSOC1*, and *HrSVP* genes related to flower organ recognition. We used GFP as a nuclear marker to verify that the *HrSEP1* gene is expressed in the nucleus. The interaction between the *HrSEP1* gene and the key enzyme genes *HrAP1*, *HrSOC1*, and *HrSVP* involved in flower development was confirmed by a yeast two-hybrid experiment and a bimolecular fluorescence complementation technique. Our observations of the transgenic plants revealed that the overexpression of the *HrSEP1* gene resulted in significant morphological changes to the flower organs. These results suggest that *HrSEP1* may have the involvement of *HrAP1*, *HrSOC1*, and *HrSVP* in the regulation of flower development. Our research provides a solid foundation for further study of the molecular basis of flower development in *H. rhamnoides* and for optimizing the germplasm resources of *H. rhamnoides*.

## Data Availability

The original contributions presented in the study are included in the article/**Supplementary Material**. Further inquiries can be directed to the corresponding authors.
