# Supplementary material for: The SEPALLATA-like gene HrSEP1 in Hippophae rhamnoides regulates flower development by interacting with other MADS-box subfamily genes
Source: Front Plant Sci. 2025 Jan 29;15:1503346. doi: 10.3389/fpls.2024.1503346 (PMC11813943; doi:10.3389/fpls.2024.1503346)
Supplement: Supplementary file 2 [file Presentation1.pptx]

## Slide 1
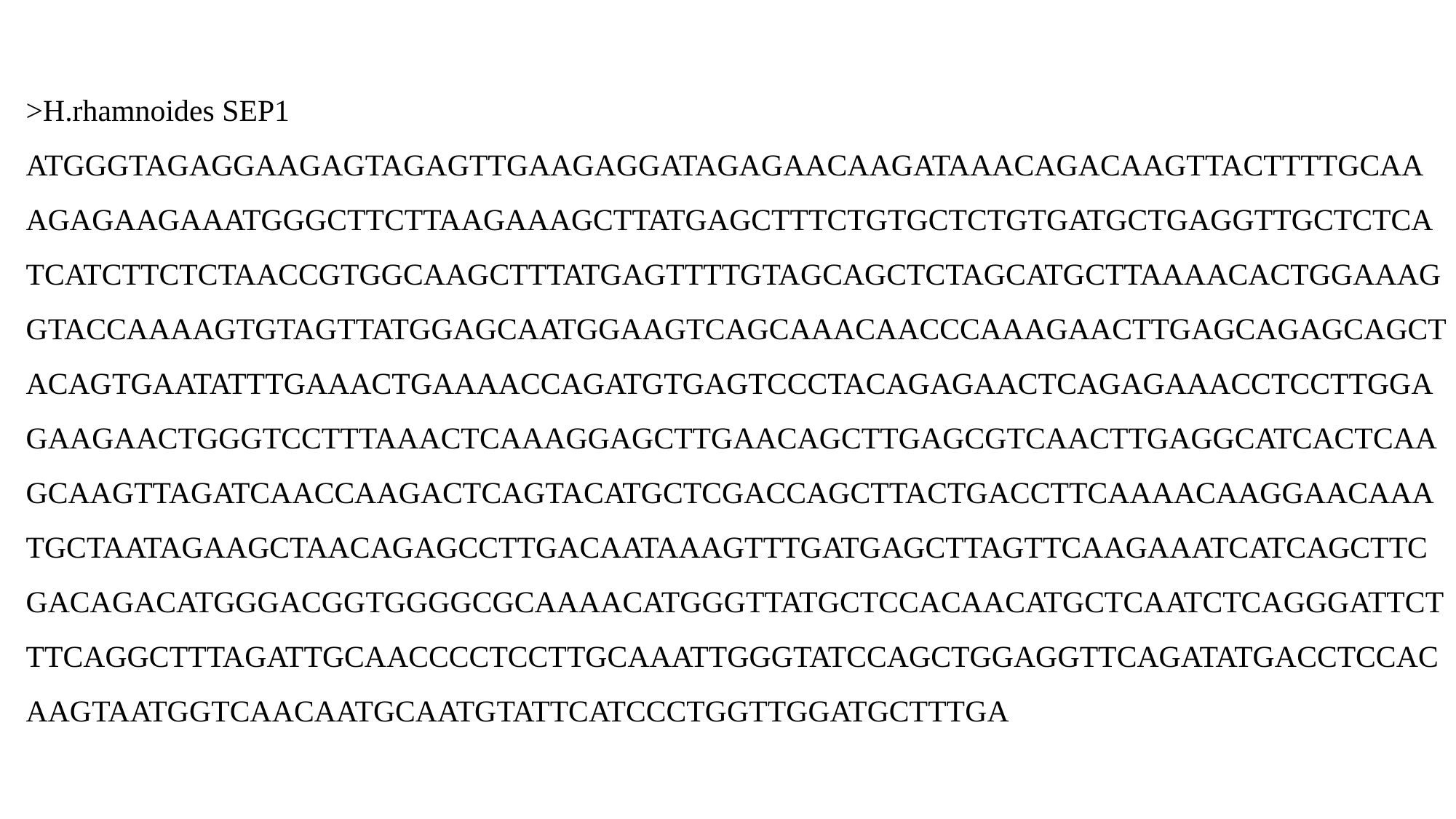

>H.rhamnoides SEP1
ATGGGTAGAGGAAGAGTAGAGTTGAAGAGGATAGAGAACAAGATAAACAGACAAGTTACTTTTGCAAAGAGAAGAAATGGGCTTCTTAAGAAAGCTTATGAGCTTTCTGTGCTCTGTGATGCTGAGGTTGCTCTCATCATCTTCTCTAACCGTGGCAAGCTTTATGAGTTTTGTAGCAGCTCTAGCATGCTTAAAACACTGGAAAGGTACCAAAAGTGTAGTTATGGAGCAATGGAAGTCAGCAAACAACCCAAAGAACTTGAGCAGAGCAGCTACAGTGAATATTTGAAACTGAAAACCAGATGTGAGTCCCTACAGAGAACTCAGAGAAACCTCCTTGGAGAAGAACTGGGTCCTTTAAACTCAAAGGAGCTTGAACAGCTTGAGCGTCAACTTGAGGCATCACTCAAGCAAGTTAGATCAACCAAGACTCAGTACATGCTCGACCAGCTTACTGACCTTCAAAACAAGGAACAAATGCTAATAGAAGCTAACAGAGCCTTGACAATAAAGTTTGATGAGCTTAGTTCAAGAAATCATCAGCTTCGACAGACATGGGACGGTGGGGCGCAAAACATGGGTTATGCTCCACAACATGCTCAATCTCAGGGATTCTTTCAGGCTTTAGATTGCAACCCCTCCTTGCAAATTGGGTATCCAGCTGGAGGTTCAGATATGACCTCCACAAGTAATGGTCAACAATGCAATGTATTCATCCCTGGTTGGATGCTTTGA
